# Ochratoxin A and β2-Microglobulin in BEN Patients and Controls

**DOI:** 10.3390/toxins2040780

**Published:** 2010-04-20

**Authors:** Pavlina Yordanova, Karmaus Wilfried, Svetla Tsolova, Plamen Dimitrov

**Affiliations:** 1 National Center of Public Health Protection, 1431 Sofia, Bulgaria; Email: p.jordanova@ncphp.government.bg (P.Y.); sv.tzolova@ncphp.government.bg (S.T.); sv.tzolova@ncphp.government.bg (P.D.); 2 Department of Epidemiology and Biostatistics, University of South Carolina, Columbia, South Carolina, USA

**Keywords:** BEN, ochratoxin A, β2-microglobulin

## Abstract

Ochratoxin A (OTA) is a mycotoxin naturally occurring in different foods. OTA is arguably a risk factor for Balkan endemic nephropathy (BEN). The aims of this study are to (1) test the OTA-BEN association in BEN-groups and controls and (2) determine whether urine β2-microglobulin, a marker of impaired ability of the kidneys to re-absorb, is related to OTA. BEN patients had significantly higher OTA serum levels. Within the offspring, OTA was significantly related to higher β2-microglobulin excretion. OTA (2005/2006) was related to a higher incidence of BEN after 2008, providing further evidence that OTA is a risk factor for BEN.

## 1. Introduction

Ochratoxin A (OTA) is a potently nephrotoxic and carcinogenic fungal metabolite, the potency varying between species and sexes, and it is also teratogenic and immunotoxic [1,2]. In 1993, the International Agency for Research on Cancer classified OTA as a possible human carcinogen (Group 2B) [1,2]. In various types of food stored under specific conditions, it is a naturally occurring contaminant, produced by *Penicillium verrucosum* and *Aspergillus* species and known as “store” fungi [2,3,4]. There is considerable information on the occurrence of OTA in cereals [2,5,6], beer [7,8], coffee beans [2,9,10], cocoa beans [11,12], dried vine fruit, wine, grape juice [2,13,14,15], and spices [16,17]. It has been demonstrated that OTA is typical contaminant of food products traditionally used by Bulgarian populations [18,19,20,21].

The ubiquitous occurrence of OTA is considered as a human health risk. Concentrations in human samples have been measured in many populations. In most European countries, the reported mean concentration of OTA in serum/plasma of healthy individuals did not exceed 1.0 ng/mL. Mean OTA concentrations in blood samples were: 0.21 ng/mL in Poland (range 1.0–13.0 ng/mL) [22] and 0.75 ng/mL in Germany (range 0.1–8.4 ng/mL) [23]. Comparable levels were detected in serum samples: 0.63 ng/mL in Czech Republic [24] and 0.56 ng/mL in Italy (range 0.12–2.84 ng/mL) [25]. Higher mean values of 1.5, 2.3 and 1.6 ng/mL in blood samples (1986, 1987, and 1988, respectively) were found in a Danish study [26]. Studies have shown that OTA is not only just a European problem, but an international. An investigation by Scott *et al*. [27], in Canada demonstrated that OTA was found in all plasma samples with a mean concentration of 0.88 ng/mL (range 0.29–2.37 ng/mL). A study in Tokyo [28] suggested that the population was frequently exposed to mycotoxin, although the levels in plasma were far less than that reported in Europe and Canada. In Lebanon OTA was detectible in 33% of human plasma samples with a mean of 0.17 ng/mL [29]. 

Ochratoxin A gained notice for human health due to the suspicion that it may be involved in the pathogenesis of Balkan endemic nephropathy (BEN) and of certain other forms of interstitial nephritis [30]. BEN is a chronic tubulointerstitial kidney disease that occurs in some areas of Bosnia and Herzegovina, Bulgaria, Croatia, Romania, Serbia, and Monte Negro [31]. To clarify the role of OTA in BEN initiation, studies in these countries focused on endemic BEN regions. Results demonstrated that OTA was more frequently found in food from endemic areas as compared to non-endemic areas [18,19,20,21,32,33]. An investigation of OTA in blood from people living in endemic and non-endemic areas in Bulgaria in 1984, 1986, 1989, and 1990, showed that OTA was present in all groups. However, it was more frequently found and at higher levels in blood samples from BEN patients compared to blood samples from non-affected people in endemic and control areas [34]. The detected OTA blood levels of healthy humans, included in this study, were in the range of 1.0–10.0 ng/mL and were supported by a later investigation of Petkova-Bocharova *et al*. [35]. Sixteen healthy volunteers who lived in two villages located in Vratza District (a high risk area for BEN in Bulgaria) had average serum concentration of 1.59 ng/mL (standard deviation: sd = 0.44). The medians were 0.4 ± 0.22 (sd = 0.22) and 0.7 (sd = 0.15) in the two villages [35]. In Croatia, the presence of OTA in the blood of inhabitants from the endemic and non-endemic regions had been monitored for 10 years. In the hyperendemic village Kaniza, the incidence of OTA in human blood was up to 4.5% (range of OTA concentrations: 2–50 ng/mL). In non-endemic villages OTA in human blood was identified in 2.4% (range: 2–10 ng/mL) [36]. In Sahel, an agricultural region of Tunisia, characterized by high incidences of chronic interstitial nephropathies of unknown etiology, highest OTA levels were found among people not affected by the nephropathy [37]. Comparing the OTA blood levels in individuals residing in BEN endemic areas with those found from areas without endemic nephropathy, there is no convincing evidence that OTA blood level may play a role in endemic nephropathy. A limitation of these studies is, however, that the role of OTA often was assessed indirectly by comparing villages and not affected patients with controls. This motivated us to test the OTA-BEN association by measuring serum OTA in BEN-patients, offspring of BEN patients and controls. In addition, we determined whether β2-microglobulin excretion, a clinical marker for nephropathy, is related to OTA serum levels.

When assessing whether a factor “A” is a risk for disease “B”, “A” is considered a risk for “B” if there is a positive association between “A” and “B”. A concern in these studies is reverse causation: “B” may be a risk for “A” and not “A” for “B”. Reverse causation results from changes due to disease characteristics or disease behavior. For instance, higher serum OTA levels may result from reduced glomerular filtration [38]. The time-structure can help to decipher what comes first. However, when linking concurrently measured biomarkers such as OTA and β2-microglobulin, a time-order cannot be established since both characteristics are measured at the same time. To avoid false interpretation, it was decided not to focus on BEN patients, but to use another approach: Adult offspring of BEN patients and control patients, the latter being admitted to the Vratza hospital, were identified. The offspring did not have yet developed the BEN, thus excluding reverse causation. To consider the time order, OTA was measured once in 2005/2006, but β2-microglobulin was determined in the same individual twice, in 2005/2006 and in 2006/2007. Linking OTA levels in 2005/2006 with β2-microglobulin in 2006/2007 addresses the time order required in the assessment of health risks. In addition, the two offspring groups were followed until 2009. Of the 38 offspring of BEN patients, 13 developed BEN themselves. This facilitated to assess the impact of OTA levels in 2005/2006 on the inception of the disease in 2009.

## 2. Materials and Methods

### 2.1. Material

Ochratoxin A analyses

#### Samples

Samples of human blood were collected in clinical laboratory in Vratza. The samples were taken in 2005 and 2006 at two time periods: 24 samples in April–July and 54 samples in October-March. All blood samples were centrifuged to separate serum and serum was stored at −20 °C. All samples were shipped frozen and stored at ≤−20 °C in the laboratory of the National Center of Public Health Protection, Sofia, until the analyses of ochratoxin A were conducted. A total of 78 serum samples were analyzed: 18 from BEN patients, 38 from BEN offspring, and 22 controls. We did not attempt to associate β_2_-microglobulin excretion in urine of BEN patients, since we were concerned that serum OTA level and β_2_-microglobulin excretion may be affected by the disease status.

#### Reagents and consumables

Crystalline ochratoxin A (CAS number 303-47-9) (Sigma-Aldrich Co.), OchraTest® (Vicam, USA) immunoaffinity columns; and chemical solvents (Merck, Germany) were of analytical or HPLC-grade quality. The following solutions were prepared: Phosphate buffered saline (PBS) (pH 7.4)—prepared by dissolving 7.0117 g NaCl, 0.2013 g KCl, 1.42 g Na_2_HPO_4_ in 1 L with purified water (adjusted pH with HCl); Ochratoxin A stock solution was prepared by dissolving 1 mg of the ochratoxin A (crystals) in 50 mL toluene and glacial acetic acid (99 + 1, V + V). The exact concentration of the stock solution was determined specrophotometrically, recording maximum absorption between a wavelength of 300 nm and 370 nm, with solvent mixture as a reference in 1 cm quartz cell (M_OTA_= 403 g/mol, ε = 544 m^2^/mL). The solution was stored at 4 °C and its stability was monitored. A series of ochratoxin A working standard solutions in mobile phase, in the range of 20, 10, 8, 6, 4 and 2 ng/mL were prepared on the day of analysis.

#### Equipment

Ochratoxin A analysis were carried out by a chromatographic system consisting of Perkin Elmer series 200 pump, Rheodine model 7125 injector valve (50 μL), and fluorescence detector Perkin Elmer 3000 (Ex = 333 nm, Em = 477 nm), analytical column—Lichrosphere 100RP18 (250 × 4.6, 5 μm, Merck, Germany).

### 2.2. Methods

#### 2.2.1. Ochratoxin A analysis in serum

The samples were analyzed for the presence of OTA by immunoaffinity column clean-up and subsequent HPLC separation with fluorescence detection based on the method of Zimmerli and Dick [39], with some modifications: we worked with OchraTest® (Vicam, USA) immunoaffinity column, instead of those used in the study of Zimmerli and Dick [39]. Briefly: 2 mL serum was extracted by 10 mL chloroform after adding 10 mL of solution containing o-phosphoric acid and NaCl [39] on Vortex. The mixture was centrifuged at 1540 g for 20 m. The clear organic phase was evaporated to dryness at 60 °C (Rotavapor). The dry residue was redissolved in 20 mL solution of PBS + methanol (75 + 15, V + V) and was loaded on the IAC. The sample extract was passed trough the IAC (ca.1–2 drops/s) using a pump stand (Vicam, USA). After washing of the IAC with 10 mL of water, the OTA was eluted with 2 mL methanol. The eluate was evaporated to dryness under a stream of nitrogen and was stored at 4 °C until HPLC determination.

##### Chromatographic conditions

For the analytical column, available in our lab, we used a more appropriate mobile phase for HPLC: acetonitrile + distilled water + glacial acetic acid (100 + 98 + 2, V + V + V). Flow rate was 1 mL/min, isocratic elution. Temperature: ambient; injection volume: 50 μL. The dry residue was carefully dissolved in 200 μL of mobile phase. 

*Method parameters*: During implementation of the HPLC method were elaborated blank and spiked samples at three different concentration levels—0.2, 0.4, 0.6 ng OTA/mL serums. The achieved recovery was 93.0% ± 10%. 

*Linearity*: Linearity was determined by a series of standards injection at six different concentrations in the range 2 ng/mL–20 ng/mL. The calibration curve showed a good linear relationship between peak height and OTA concentration with coefficient of correlation R^2^ > 0.999.

*Limit of detection (LOD):* with the described procedure the detection limit was 0.1 ng/mL, based on 3:1 signal to noise ratio.

Calculated RSD, on the base of spiked samples was 5%.

#### 2.2.2. β2-microglobulin in urine

β2-microglobulin in urine is a marker of impaired inability of the kidneys to re-absorb. Briefly, urine collected during the first hour of the 4-hour period [40], was analyzed for β_2_-microglobulin using a chemiluminescent immunoassay (Cat. No.LKBM1, DPC, USA) with the Immulite analyzer (DPC, USA). 

##### Statistical Methods

Because the distributions of serum-OTA and β2-microglobulin in urine were not normal, we present their medians, conducted log-transformations, and used geometric means and their confidence limits. To answer the first question, whether BEN patients and offspring of BEN parents had higher serum OTA levels, we compared three groups (BEN patients, offspring of BEN patients, and offspring of control patients) using linear regression analyses (PROC GLM) [41]. Controlling for age, sex of the participant, and season of sample collection, we estimated the adjusted geometric means of the three groups. To answer the second question, whether β2-microglobulin in urine, a marker for kidney function [40] that was measured in offspring only, is related to OTA in serum, we also applied linear regression. After testing linearity, age was treated a continuous predictor of β2-microglobulin. Month of sample collection was grouped into “October to March” and “April to July”. Statistically controlling for age, sex of the participant, and season of sample collection, linear regression analyses provided the slope (parameter estimate) between OTA exposure and β2-microglobulin. In addition, to compare the extent to which β2-microglobulin measured in 2005/2006 and 2006/2007 were correlated we estimated Spearman rank correlations. Finally, we compared OTA serum levels measured in 2005/2006 in disease-free adult offspring; some of them later, after 2008, definitely or possibly developed BEN. All statistical analyses were performed using SAS 9.2 [41]. 

## 3. Results and Discussion

### 3.1. Results

#### 3.1.1. Population

OTA serum levels were measured in 78 participants in 2005/2006; 18 were BEN patients, 38 offspring of BEN patients and 22 were offspring of hospital controls who had other diseases (Table 1). BEN patients were approximately 20 years older (72.5 years) than the adult offspring of BEN and non-BEN patients (controls). In addition, in the offspring group with OTA serum determinations (n = 60: 38 + 22), β2-microglobulin levels were measured in 59 subjects in 2005/2006 (37 BEN and 22 control offspring) and again in 58 subjects in 2006/2007 (36 BEN and 22 control offspring).

#### 3.1.2. Ochratoxin A and β_2_-microglobulin analysis

**Table 1:** Population characteristics.

	BEN patients	Offspring of BEN patients	Offspring of controls patients
	n = 18	n = 38	n = 22
**Age in years**, median (5%–95% values)	72.5 (61.0–79.0)	49.0 (38.0–63.0)	51.0 (35.0–61.0)
**Gender, % (male)**	55.6 (10/18)	57.9 (22/38)	63.6 (14/22)
**Months of collection**			
April–July, %	22.2	50	4.5
October–March, %	78.8	50	95.5
**Ochratoxin A in serum** (ng/mL) median (5–95% values)	1.15 (0.40–3.90)	0.70 (0.30–1.80)	0.56 (0.39–0.93)
**β2–microglobulin in urine2005/2006** (ng/mL), median (5%–95% values)	-	51.00 (12.30–500.00)	50.25 (22.40–152.00)
**β2–microglobulin in urine2006/2007** (ng/mL), median (5%–95% values)	-	56.55 (14.60–500.00)	34.75 (14.90–156.00)

By chance, only few samples from the offspring of the control patients were collected between April and July. In two families, both BEN patients and offspring participated. However, they were not living together in the same household and their values did not show a substantial agreement (family 1: BEN patient: 3.9 ng OTA/mL serum; offspring: 0.7 ng OTA/mL serum; family 2: BEN patient: 3.1 ng OTA/mL serum; offspring: 1.4 ng OTA/mL serum). For this reason, we did not adjust for coming from the same family.

All of the tested BEN patients had detectible OTA serum levels, and the values are in the range of 0.4 ng/mL to 3.9 ng/mL, with median of 1.15 ng/mL (adjusted geometric mean: 1.19 ng/mL, Table 2). Serum OTA was also significantly higher in BEN patients compared to adult offspring of BEN and non-BEN patients. The highest OTA level found was 3.9 ng/mL in the serum of a BEN patient. In the group of BEN offspring, the median was 0.70 ng/mL (adjusted geometric mean: 0.76 ng/mL), and only one sample is <LOD (0.1 ng/mL). In controls the median was 0.56 ng/mL (adjusted geometric mean: 0.72 ng/mL), and two participants had OTA level <LOD. The season of serum collection affected the serum OTA concentration (Table 2). It was higher in samples collected in March to July. 

**Table 2:** Adjusted geometric means for ochratoxin A derived from linear regression analyses ^#^.

	Geometric mean ng/mL	5%–95% value ng/mL	P-value (t-test)
Season			
(March–July)	1.09	0.86–1.37	0.001
(October–February)	0.69	0.61–0.79	
BEN patients (n = 18)	1.19	0.86–1.66	0.02
Offspring of BEN patients (n = 38)	0.76	0.64–0.89	0.73
Offspring of controls (n = 22)	0.72	0.56–0.92	reference
^#^ Adjusted for all variables above and age and sex of the participant.			

The analysis of whether β2-microglobulin excretion is related to OTA focused on the group of adult offspring of BEN and non-BEN patients (Table 3). Complete data for all variables was available for 56 of 59 participants in 2005/2006 and 55 of 58 in 2006/2007. The results show that serum OTA collected in 2005/2006 was significantly positively associated with β2-microglobulin in the same year and one year later. β2-microglobulin excretion was increased when participants were older. Sex of the participant and month of collection did not affect this association. The two measurements of β2-microglobulin in 2005/2006 and 55 of 58 in 2006/2007 were correlated (Spearman rank correlation coefficient: 0.55, p = 0.0001, n = 58), which indicate some stability over time.

**Table 3:** Results of linear regression analyses.

Parameter estimates explaining log10 of β_2_–microglobulin in urine (ng/mL) ^#^				
	2005–2006 (n = 56)		2006–2007 (n = 55)	
Predictor	Parameter estimated	P-value	Parameter estimated	P-value
Serum OTA, (ng/mL)	0.49	0.002	0.36	0.0496
Age (per year)	0.016	0.01	0.019	0.01
Sex (female)	−0.014	0.18	−0.017	0.89
April–July	−0.17	0.14	0.08	0.56
^# ^Mutually adjusted for all variables above.				

Since the risk of developing BEN is considered to be much higher in offspring of BEN patents, we followed offspring of BEN patients and offspring of control patients with yearly examination after 2008. Of the 60 offspring (Table 1), we have information on 57 participants (38 offspring of BEN patients and 22 offspring of control patients). Of the initially BEN-free offspring, 13 developed BEN (six definite and seven suspected cases). OTA serum concentration showed a dose-response relation with the likelihood of BEN (no, suspected, definite; Table 4). Higher levels of OTA in 2005/2006 were related to an increased certainty of BEN incidence (p = 0.0012). 

**Table 4:** .Ochratoxin A in serum of participants in 2005/2006 and incidence of Balkan Endemic Nephropathy in 2009.

Serum Ochratoxin A (OTA) levels in disease-free and incident cases						
	Non BEN		Suspected BEN		Definite BEN	
	n = 44		n = 7		n = 6	
**Exposure**	**median**	**5%–95% values**	**median**	**5%–95% values**	**median**	**5%–95% values**
Serum OTA in 2005/2006 (ng/mL)	0.60	0.33–1.0	0.94	0.46–1.7	1.15	0.67–2.0
Kruskal-Wallis test: P-value = 0.0012.						

### 3.2. Discussion

Our findings show that ochratoxin A serum levels were significantly higher in BEN patients compared to offspring of BEN patients and offspring of control patients. Within a population of adult offspring of BEN and non-BEN patients, OTA was statistically significantly related to higher β_2_-microglobulin excretion in urine. The association was not restricted to a concurrent effect (both markers measured at the same time) but also showed delayed affect. Hence, it is possible that OTA exposure may have a long-term toxic effect on the kidneys [2]. Although in 2005/2006, the serum concentration of OTA did not differ between offspring of BEN patients and offspring of control patients, OTA levels in 2005/2006 were associated with the incidence of BEN after 2008. 

Limitations are that the age of BEN patients, given the occurrence of BEN later in life, is higher (72.5 years) than the age of offspring of BEN patients and offspring of control patients (approximately 50 years). However, also controlling for age, OTA levels were significantly higher in BEN patients. Another limitation is uneven distribution of OTA samples during the year. We were surprised to find serum OTA levels in participants were higher during March to July compared to October to February. Seasonal variability with higher OTA levels in blood serum/plasma in summer has been demonstrated by studies conducted in Italy, Croatia, and Spain [25,42,43,44]. In Tuscany, Italy, the OTA serum levels found in October were 34% lower than those found in July. In Croatia the levels found in December were 51% lower than those found in June. The finding that OTA levels were higher in summer and lower in winter may reflect changes in conditions favoring mould growth and toxin production [45]. Additionally, the period of time between harvesting and consumption of cereals may contribute to seasonal variation in OTA exposure. Cereals consumed during June will probably have been stored since the previous year, during which time the growth of OTA producing mould species can occur. In contrast, cereals consumed between September and December is likely to have been harvested more recently and is therefore a lower risk for OTA contamination [45]. Nevertheless, we adjusted the OTA levels for the effect of season and thus minimized this bias.

The analytical parameters of the method for OTA determination, implemented in our laboratory (LOD 0.1 ng/mL, recovery 93%) are comparable with recent methods used in this field. The immunoaffinity clean-up and the HPLC separation and fluorescence detection give the advantage of sensitivity and specificity. 

Regarding serum OTA, our findings support the results of an earlier study performed in Bulgaria [34]. However, the highest value found in our study (3.9 ng/mL in one BEN patient) is only half of the highest value found in the study of Petkova-Bocharova *et al*. [35] in the healthy people residing in the same region in which our study was conducted. Comparing our results to a Croatian investigation that focused on the endemic village Kaniza and some non-endemic villages, our result are closer to the lowest value found in non-endemic villages (2 ng/mL) [36]. In addition, the levels we measured are comparable to levels reported in other European countries, excluding the higher values found in Poland (13.0 ng/mL) [22] and in Italy (57.2 ng/mL) [25]. 

Within the adult offspring of BEN and non-BEN patients, OTA was statistically significantly related to higher β_2_-microglobulin excretion in urine. A higher β2-microglobulin is considered a marker of reduced kidney function [40]. Hassen *et al*. [46], conducted an investigation in Tunisia, a region considered to have higher OTA exposure, in patients with chronic interstitial nephropathy. The investigators did not directly linked OTA and ß2-microglobulin, but showed that both are higher in spot urine samples of patients with chronic kidney diseases [46]. In two healthy control groups, the mean OTA levels were 2.6 ng/mL and 1.2 ng/mL; in disease patients the mean OTA levels were 44.4, 8.11, 50.4, and 12.4 ng/mL. The median OTA levels in our study were 1.15 ng/mL for BEN patients, and 0.7 ng/mL for BEN offspring. In two healthy control groups in the Tunisian study, the mean ß2-microglobulin levels were 90 ng/mL and 120 ng/mL; in diseased patients the mean ß2-microglobulin levels were 2350, 710, 1960, and 1440 ng/mL. The median ß2-microglobulin level in our study were about 51–56 ng/mL for BEN offspring. We cannot explain why OTA, ß2-microglobulin and levels found by Hassen *et al.* [46] in an agricultural region of Tunisia were much higher than the levels in our study. It is possible, that to some extent the higher levels in the Tunisian study are due to use of means instead of medians in the case of skewed OTA distributions.

In addition, an Egyptian study on OTA in both human milk and infant serum showed a statistical significant association between OTA levels in maternal milk and β_2_-microglobulin in serum of their infants [47]. ß2-microglobulin levels in infants of OTA positive mothers (n = 36) were 260 µg/mL and 30 µg/mL in infants of mothers without detectable OTA concentration (n = 14). The difference was not statistically significant different when the investigators applied logistic regression analyses and controlled for other factors.

Comparing a high risk group of adult offspring from parents who had BEN and a group of offspring of control patients without history of BEN, our follow-up study demonstrated for the first time, that serum concentration of OTA may contribute to the development of BEN. In adult offspring, who did develop BEN in 2009, OTA levels in 2005/2006 were twice the level of those who did not develop the disease. Since there were no differences in the two offspring groups in 2005/2006 and since all new BEN cases occurred in offspring with a family history, it is likely the OTA does not act alone but in combination with family predisposition. 

Although attention has focused on the OTA-BEN association, other nephrotoxic mycotoxins should not be ignored. For instance citrinin, a nephrotoxic mycotoxin produced by *Aspergillus*, *Penicillum* and *Monascus* spp., has been given comparatively less consideration. However, it is similar to OTA in its toxicology and both toxins may have additive or synergistic effects [48,49,50,51]. Citrinin was found in food sampled in the BEN area in Bulgaria [18]. Later, in this area, contamination of food by high levels of citrinin was confirmed in a study by Vrabcheva *et al.* [19]. Summarizing these data, Pfohl-Leszkowicz *et al.* [52] noted that areas in Bulgaria affected by BEN are characterized by a more frequent exposure to both OTA and citrinin. 

## 4. Conclusions

Our study provides evidence that OTA levels pose a risk for developing the disease and are higher in BEN patients. The observation that the season of sample collection may affect the serum OTA concentration needs to be taken into account in the future studies. In particular, results form regional comparisons may be compromised by seasonal effects. The reported association between OTA and β2-microglobulin was not restricted to a concurrent but also showed a delayed effect and additionally was observed in one other study. However, to better understand the role of mycotoxins in the pathogenesis of BEN, future studies should not only investigate ochratoxin A, but also citrinin.

## References

[1] Walker R. (2002). Risk assessment of ochratoxin: Current views of the European Scientific Committee on food, the JECFA and the Codex Committee on food additives and contaminants. Adv. Exp. Med. Biol..

[2] Benford D., Boyle C., Dekant W., Fuchs R., Gaylor D., Hard G., McGregor D., Pitt J., Plestina R., Shephard G., Solfrizzo M., Verger Ph., Walker R.  (2001). Ochratoxin A. Safety Evaluation of Certain Mycotoxins in Food.

[3] Abarca M.L., Accensi F., Bragulat M.R., Cabaňes F.J. (2001). Current importance of ochratoxin A-producing *Aspergillus* spp.. J. Food Prot..

[4] Magan N., Aldred D. (2005). Conditions of formation of ochratoxin A in drying, transport and in different commodities. Food Addit. Contam..

[5] Beretta B., de Domenico R., Gaiaschi A., Ballabio C., Galli C.L., Gigliotti C., Restani P. (2002). Ochratoxin A in cereal-based baby foods: occurrence and safety evaluation. Food Addit. Contam..

[6] Biffi R., Munari M., Dioguardi L., Ballabio C., Cattaneo A., Galli C.L., Restani P. (2004). Ochratoxin A in conventional and organic cereal derivatives: A survey of the Italian market, 2001–2002. Food Addit. Contam..

[7] Tangni E.K., Ponchaut S., Maudoux M., Rozenberg R., Larondelle Y. (2002). Ochratoxin A in domestic and imported beers in Belgium: Occurrence and exposure assessment. Food Addit. Contam..

[8] Medina A., Jiménez M., Gimeno-Adelantado J.V., Valle-Algarra F.M., Mateo R. (2005). Determination of ochratoxin A in beer marketed in Spain by liquid chromatography with fluorescence detection using lead hydroxyacetate as a clean-up agent. J. Chromatogr. A.

[9] Otteneder H., Majerus P. (2001). Ochratoxin A (OTA) in coffee: Nation-wide evaluation of data collected by German Food Control 1995–1999. Food Addit. Contam..

[10] Lombaert G.A., Pellaers P., Chettiar M., Lavalee D., Scott P.M., Lau B.P. (2002). Survey of Canadian retail coffees for ochratoxin A. Food Addit. Contam..

[11] Amézqueta S., González-Peňas E., Murillo M., de Cerain A.L. (2005). Occurrence of ochratoxin A in cocoa beans: Effect of shelling. Food Addit. Contam..

[12] Jørgensen K. (2005). Occurrence of ochratoxin A in commodities and processed food─a review of EU occurrence data. Food Addit. Contam..

[13] Cabaňes F.J., Accensi F., Bragulat M.R., Abarca M.L., Castellá G., Minguez S., Pons A. (2002). What is the source of ochratoxin A in wine?. Int. J. Food Microbiol..

[14] Abarca M.L., Accensi F., Bragulat M.R., Castellá G., Cabaňes F.J. (2003). *Aspergillus carbonarius* as the main source of ochratoxin A contamination in dried vine fruits from the Spanish market. J. Food Prot..

[15] Battilani P., Pietri A., Bertuzzi T., Languasco L., Giorni P., Kozakiewicz Z. (2003). Occurrence of ochratoxin A-producing fungi in grapes grown in Italy. J. Food Prot..

[16] Thirumala-Devi K., Mayo M.A., Reddy G., Tangni E.K., Larondelle Y., Reddy D.V. (2001). Occurrence of ochratoxin A in black pepper, coriander, ginger and turmeric in India. Food Addit. Contam..

[17] Fazekas B., Tar A., Kovács M. (2005). Aflatoxin and ochratoxin A content of spices in Hungary. Food Addit. Contam..

[18] Petkova-Bocharova T., Castegnaro M., Michelon J., Maru V., Castegnaro M., Pleština R., Dirheimer G., Chernozemsky I.N., Bartsch H. (1991). Ochratoxin A and other mycotoxins in cereals from an area of Balkan endemic nephropathy and urinary tract tumours in Bulgaria. Mycotoxins: Endemic Nephropathy and Urinary Tract Tumours.

[19] Vrabcheva T., Usleber E., Dietrich R., Märtlbauer E. (2000). Co-occurrence of ochratoxin A and citrinin in cereals from Bulgarian villages with a history of Balkan endemic nephropathy. J. Agric. Food Chem..

[20] Abouzied M.M., Horvath A.D., Podlesny P.M., Regina N.P., Metodiev V.D., Kamenova-Tozeva R.M., Niagolova N.D., Stein A.D., Petropoulos E.A., Ganev V.S. (2002). Ochratoxin A concentrations in food and feed from a region with Balkan Endemic Nephropathy. Food Addit. Contam..

[21] Vrabcheva T., Petkova-Bocharova T., Grosso F., Nikolov I., Chernozemsky I.N., Castegnaro M., Dragacci S. (2004). Analysis of ochratoxin A in foods consumed by inhabitants from an area with balkan endemic nephropathy: A 1 month follow-up study. J. Agric. Food Chem..

[22] Golinski P. (1987). Ochratoxin A in human organism as a result of food and feed contamination. Rocz. AR Poznaniu..

[23] Hadlok R.M., Creppy E.E., Castegnaro M., Dirheimer G. (1993). Human ochratoxicosis in Germany updating 1993. Human Ochratoxicosis and its Pathologies.

[24] Ruprich J., Ostry V. (1993). Health risk assessment of the mycotoxin ochratoxin A to humans: Czech Republic–Brno–1991/92. Cent. Eur. J. Public Health.

[25] Palli D., Miraglia M., Saieva C., Masala G., Cava E., Colatosti M., Corsi A.M., Russo A., Brera C. (1999). Serum levels of ochratoxin A in healthy adults in Tuscany: Correlation with individual characteristics and between repeat measurements. Cancer Epidemiol. Biomarkers Prev..

[26] Hald B., Castegnaro M., Pleština R., Dirheimer G., Chernozemsky I.N., Bartsch H. (1991). Ochratoxin A in human blood in European countries. Mycotoxins, Endemic Nephropathy and Urinary Tract Tumours.

[27] Scott P.M., Kanhere S.R., Lau B.P.Y., Lewis D.A., Hayward S., Ryan J.J., Kuiper-Goodman T. (1998). Survey of Canadian human blood plasma for ochratoxin A. Food Addit. contam..

[28] Ueno Y., Maki S., Lin J., Furuya M., Sugiura Y., Kawamura O. (1998). A 4-year study of plasma ochratoxin A in a selected population in Tokyo by immunoassay and immunoaffinity column-linked HPLC. Food Chem. Toxicol..

[29] Assaf H., Betbeder A.M., Creppy E.E., Pallardy M., Azouri H. (2004). Ochratoxin A levels in human plasma and foods in Lebanon. Hum. Exp. Toxicol..

[30] Gekle M., Sauvant C., Schwerdt G. (2005). Ochratoxin A at nanomolar concentrations: A signal modulator in renal cells. Mol. Nutr. Food Res..

[31] Fuchs R., Peraica M. (2005). Ochratoxin A in human kidney diseases. Food Addit. Contam..

[32] Puntarić D., Bošnir J., Šmit Z., Škes I., Baklaić Z. (2001). Ochratoxin A in corn and wheat: Geographical association with endemic nephropathy. Croat. Med. J..

[33] Pfohl-Leszkowicz A., Tozlovanu M., Manderville R., Peraica M., Castegnaro M., Stefanovic V. (2007). New molecular and field evidences for the implication of mycotoxins but not aristolochic acid in human nephropathy and urinary tract tumor. Mol. Nutr. Food Res..

[34] Petkova-Bocharova T., Castegnaro M., Castegnaro M., Pleština R., Dirheimer G., Chernozemsky I.N., Bartsch H. (1991). Ochratoxin A in human blood in relation to Balkan endemic nephropathy and urinary tract tumours in Bulgaria. Mycotoxins:Endemic Nephropathy and Urinary Tract Tumours.

[35] Petkova-Bocharova T., Castegnaro M., Pfohl-Leszkowicz A., Garren L., Grosso F., Nikolov I., Vrabcheva T., Dragacci S., Chernozemsky I.N. (2003). Analysis of Ochratoxin A in serum and urine of inhabitants from an area with Balkan Endemic Nephropathy: A one month follow up study. Facta Univer..

[36] Radić B., Fuchs R., Peraica M., Lucić A. (1997). Ochratoxin A in human sera in the area with endemic nephropathy in Croatia. Toxicol. Lett..

[37] Grosso F., Said S., Mabrouk I., Fremy J.M., Castegnaro M., Jemmali M., Dragacci S. (2003). New data on the occurrence of ochratoxin A in human sera from patients affected or not by renal diseases in Tunisia. Food Chem. Toxicol..

[38] Özçelik N., Koşar A., Soysal D. (2001). Ochratoxin A in human serum samples collected in Ispatra-Turkey from healthy individuals and individuals suffering from different urinary disorders. Toxicol. Lett..

[39] Zimmerli B., Dick R. (1995). Determination of ochratoxin A at the ppt level in human blood, serum, milk and some foodstuffs by high-performance liquid chromatography with enhanced fluorescence detection and immunoaffinity column cleanup: Methodology and Swiss data. J. Chromatogr. B Biomed. Appl..

[40] Dimitrov P., Tsolova S., Georgieva R., Bozhilova D., Simeonov V., Bonev A., Karmaus W. (2006). Clinical markers in adult offspring of families with and without Balkan Endemic Nephropathy. Kidney Int..

[41] SAS Institute (2009). SAS/STAT Software V9.2.

[42] Peraica M., Domijan A.M., Matašin M., Lucić A., Radić B., Delaš F., Horvat M., Bosanac I., Balija M., Grgičević D. (2001). Variations of ohratoxin A concentration in the blood of healthy populations in some Croation cities. Arch. Toxicol..

[43] Domijan A.M., Peraica M., Fuchs R., Lucić A., Radić B., Balija M., Bosanac I., Grgičević D. (1999). Ochratoxin A in blood of healthy population in Zagreb. Arch. Indust. Hug. Toxicol..

[44] Burdaspal P.A., Legarda T.M. (1998). Datos sobre la presencia de ochratoxin A en plasma humano en Espaňa. Alimentaria.

[45] Aish J.L., Rippon E.H., Barlow T., Hatterslay S.J., Magan N., Olsen M. (2004). Ochratoxin A. Mycotoxins in Food Detection and Control.

[46] Hassen W., Abid S., Achour A., Creppy E., Bacha H. (2004). Ochratoxin A and beta2-microglobulinuria in healthy individuals and in chronic interstitial nephropathy patients in the centre of Tunisia: A hot spot of Ochratoxin A exposure. Toxicology.

[47] Hassan A.M., Sheashaa H.A., Fattah M.F., Ibrahim A.Z., Gaber O.A., Sobh M.A. (2006). Study of ochratoxin A as an environmental risk that causes renal injury in breast-fed Egyptian infants. Pediatr. Nephrol..

[48] Kanisawa M. (1984). Synergistic effect of citrinin on hepatorenal carcinogenesis of ochratoxin A in mice. Dev. Food Sci..

[49] Kitchen D.N., Carlton W.W., Hinsman J. (1977). Ochratoxin A and citrinin induced nephrosis in beagle dogs. III. Terminal renal ultrastructural alterations. Vet. Pathol..

[50] Pfohl-Leszkowicz A., Molinié A., Tozlovanu M., Manderville R., Siantar D., Trucksess M., Scott P., Herman E. (2008). Combined toxic effects of ochratoxin A and citrinin, *in vitro* and *in vivo*. Food Contaminants; Mycotoxins and Food Allergen.

[51] Pfohl-Leszkowicz A., Manderville R. (2007). Review on Ochratoxin A: An overview on toxicity and carcinogenicity in animals and humans. Mol. Nutr. Food Res..

[52] Pfohl-Leszkowicz A., Petkova-Bocharova T., Chernozemski I.N., Castegnaro M. (2002). Balkan endemic nephropathy and associated urinary tract tumours: A review on aetiological causes and the potential role of mycotoxins. Food Add. Contam..

